# LSM-W^2^: laser scanning microscopy worker for wheat leaf surface morphology

**DOI:** 10.1186/s12918-019-0689-8

**Published:** 2019-03-05

**Authors:** Ulyana S. Zubairova, Pavel Yu. Verman, Polina A. Oshchepkova, Alina S. Elsukova, Alexey V. Doroshkov

**Affiliations:** 1 Institute of Cytology and Genetics SB RAS, Prospekt Lavrentyeva 10, Novosibirsk, 630090 Russia; 2 A.P. Ershov Institute of Informatics Systems SB RAS, Prospekt Lavrentyeva 6, Novosibirsk, 630090 Russia; 3 Novosibirsk State University, Pirogova str. 1, Novosibirsk, 630090 Russia

**Keywords:** ImageJ plugin, Confocal laser scanning microscopy, Leaf epidermal pattern, Image processing, Cereals, stomata, Pavement cells, Growth zone

## Abstract

**Background:**

Microscopic images are widely used in plant biology as an essential source of information on morphometric characteristics of the cells and the topological characteristics of cellular tissue pattern due to modern computer vision algorithms. High-resolution 3D confocal images allow extracting quantitative characteristics describing the cell structure of leaf epidermis. For some issues in the study of cereal leaves development, it is required to apply the staining techniques with fluorescent dyes and to scan rather large fragments consisting of several frames. We aimed to develop a tool for processing multi-frame multi-channel 3D images obtained from confocal laser scanning microscopy and taking into account the peculiarities of the cereal leaves staining.

**Results:**

We elaborated an ImageJ-plugin LSM-W^2^ that allows extracting data on **L**eaf **S**urface **M**orphology from **L**aser **S**canning **M**icroscopy images. The plugin is a crucial link in a workflow for obtaining data on structural properties of leaf epidermis and morphological properties of epidermal cells. It allows converting large lsm-files (laser scanning microscopy) into segmented 2D/3D images or tables with data on cells and/or nuclei sizes. In the article, we also represent some case studies showing the plugin application for solving biological tasks. Namely the plugin is applied in the following cases: defining parameters of jigsaw-puzzle pattern for maize leaf epidermal cells, analysis of the pavement cells morphological parameters for the mature wheat leaf grown under control and water deficit conditions, initiation of cell longitudinal rows, and detection of guard mother cells emergence at the initial stages of the stomatal morphogenesis in the growth zone of a wheat leaf.

**Conclusion:**

The proposed plugin is efficient for high-throughput analysis of cellular architecture for cereal leaf epidermis. The workflow implies using inexpensive and rapid sample preparation and does not require the applying of transgenesis and reporter genetic structures expanding the range of species and varieties to study. Obtained characteristics of the cell structure and patterns further could act as a basis for the development and verification for spatial models of plant tissues formation mechanisms accounting for structural features of cereal leaves.

**Availability:**

The implementation of this workflow is available as an ImageJ plugin distributed as a part of the Fiji project (FijiisjustImageJ: https://fiji.sc/). The plugin is freely available at https://imagej.net/LSM_Worker, https://github.com/JmanJ/LSM_Worker and http://pixie.bionet.nsc.ru/LSM_WORKER/.

**Electronic supplementary material:**

The online version of this article (10.1186/s12918-019-0689-8) contains supplementary material, which is available to authorized users.

## Background

Approaches to plants phenotyping based on image processing provide the basis for many current and challenging tasks that require precise classification of objects based on analysis of a large number of features. Microscopic images are widely used as an essential source of information on cells morphometric characteristics and cellular tissue architecture due to modern computer vision algorithms of segmentation [1]. Such issues are mentioned in scientific papers where images are used as a source of data for studying patterning of multiple cell types in the plant epidermis [2], investigating the topology of pavement cells for developing Arabidopsis leaves [3] and mechanisms for puzzle-like cells emerging [4], detecting exit from proliferation during Arabidopsis leaf development [5].

The epidermis of cereals leaf is a complex tissue consisting of different cell types forming a specific cellular pattern from parallel cell longitudinal rows. Occurring for a long time a unidirectional growth of these leaves enables to observe a series of successive morphogenetic stages at one time moment. Therefore, leaf epidermis is a fruitful biological model for studying mechanisms of plant morphogenesis, for example, in stressful conditions [6]. Critical in this methodology is to quantify cell geometry or other features of epidermal tissue for extended fragments of leaves. Confocal microscopy is an appropriate tool to take the quantitative characteristics describing the cellular structure of the leaf epidermis. However, involving large data-sets in the analysis requires high-performance computer image processing methods.

The plant-image-analysis database [7–9] provides an overview of existing software for plant image analysis. Among others, there are some tools suitable for quantifying properties of cells and tissues for cereal leaves accounting their structural features. The following programs are independent software systems. *MorphoGraphX* [10] and *ACME* (Automated Cell Morphology Extractor) [11] are multi-task plant tissue phenotyping tools used in various research groups to investigate growth mechanisms in both plant and animal systems. *iRoCS Toolbox* [12, 13] is developed for the analysis of the cell structure of Arabidopsis root and automatically fits standardized coordinates to raw 3D image data. *CellSeT* [14] is intended for root analysis and is not suitable for the case of the epidermis of a leaf of cereals when the pattern contains large and small neighboring cells. *Areoana* [15] allows quantifying parameters of leaf cells for the moss and is specially designed for these species. Another group of programs is implemented in the form of ImageJ (Fiji) plugins [16] that in most cases allows using multiple plugins and built-in functions within one image processing workflow. To work with images in lsm-format (laser scanning microscopy) an *LSM toolbox* [17] was developed. A plugin for stitching confocal images [18] works on 2D and 3D images. *PaCeQuant* [19] was elaborated for structural features quantification from 2D images of Arabidopsis leaves. *Morphological Segmentation* [20] implements the algorithm of marker watershed and allows to segment biological objects on images. *LobeFinder* [21] implements a convex-hull based algorithm to identify lobes, quantifies geometric properties, and creates a useful graphical output for further analysis. *Costanza* (COnfocal STack ANalyZer Application) [22] is a plugin for segmentation and analyzing stacks of image data designed for shoot apical meristem of Arabidopsis mutants expressing the green fluorescent protein on cell membranes.

Our study aimed to develop a workflow for quantifying structural properties of cereal leaves epidermis. A crucial link in this workflow is a Fiji plugin LSM-W^2^ that extracts Leaf Surface Morphology from Laser Scanning Microscopy images. The plugin is able to process multi-channel multi-frame 3D images in lsm-format obtained from confocal laser scanning microscope. During processing, the plugin takes into account structural, staining and microscopy features of the tissue studied. In the article, we describe the plugin implementation and discuss four case studies demonstrating the plugin application for solving biological tasks. Experimental images of leaf fragments were obtained from wheat (*Triticum aestivum* L.) cultivars Chinese spring, Rodina, and Saratovskaya 29, and maize (*Zea mays* L.) inbred line 611 originated from cv. Mo17.

## Implementation

### Technique for 3D images obtaining

For successful segmentation, on the input images, the cell walls and nuclei of the epidermal cells of the leaf should be well distinguishable, and the background signal should be as low as possible. This purpose was achieved by staining of fixed samples of leaves fragments with a set of fluorescent dyes (DAPI and PI for leaf fragments from the mature zone, CW and PI for leaf fragments from the growth zone). Additional file 1 contains a detailed sample preparation protocol. The resulted 3D images visualized cell walls in the “blue” channel and the cell nuclei in the “red” channel.

A laser scanning microscope LSM 780 NLO (Zeiss, Germany) based on AxioObserver Z1 (Zeiss, Germany) was used to obtain 3D images. A 3D scan of an extended region in high resolution (more than 1 pixel per 1 *μ*m) allows quantifying characteristics of the leaf epidermal cellular pattern (size and shape of cells and nuclei, and their mutual arrangement). A relatively small view field of the microscope compared with the size of the required leaf fragment resulted in repetitive movements of the scan area (“tile scan” mode). Note that it is necessary to provide such resolution along the vertical axis also. To scan cereal leaves we used from 40 to 150 z-slices, depending on the degree of the leaf surface curvature. For the correct segmentation, it is necessary that the thickness of the cell wall and the nucleus diameter consist of more than about 3-5 pixels. Hence, by estimating the average sizes of large and small cells (for growing and mature zones of leaves), parameters of magnification and the distance between neighboring optical slices should be set correctly. Thus, the obtained 3D-images are multi-frame and multi-channel (two channels were used in this work) lsm-files. Each frame in each color channel consists of a set of square-shaped slices (as a rule their size is 512 × 512, 1024 × 1024, or 2048 × 2048 pixels) packed in a stack.

### Processing of 3D images with LSM-W^2^

A 3D-image obtained according to the described above method must be downloaded into the plugin. The output can be obtained as segmented images where the areas of cells and nuclei are marked as well as the results of measurements and comparisons of the volumes of cells and nuclei in table form. A segmented image is a tif-file in which each pixel can have one of the following values: 0 (background), 1 (segment boundary) or a unique index of the segment (cell or nucleus).

**Fig. 1:**
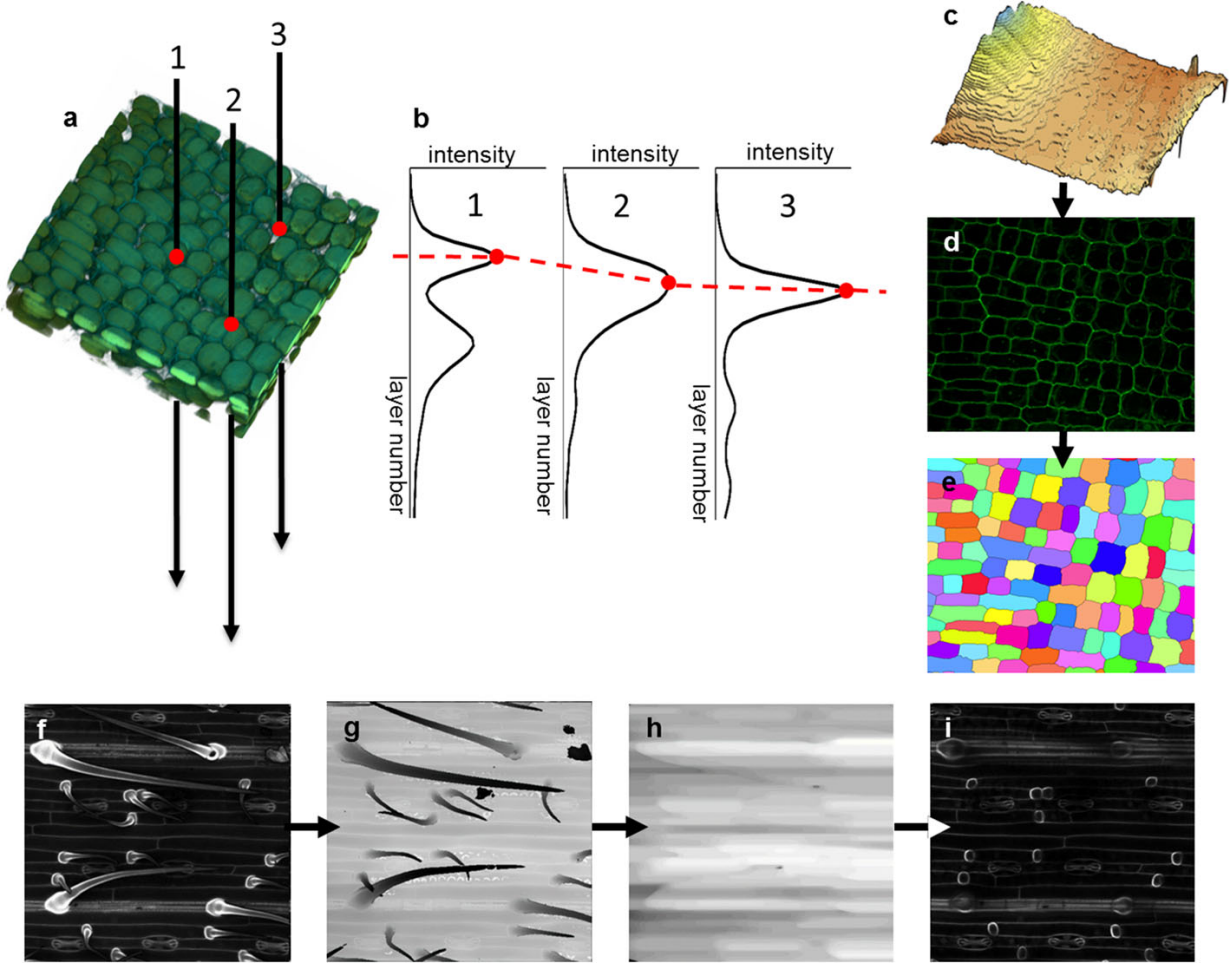
Formation of a virtual cut reflecting the cellular structure of the epidermal layer by LSM-W^2^ plugin. **a-e** Construction of the leaf surface and 2D virtual cut. **a** Fragment of 3D reconstruction of the wheat leaf epidermis in the growth zone. Points 1, 2, 3 denote virtual punctures; red dots indicate the leaf surface. **b** Plots of intensity vs. slice number corresponding to three virtual punctures from A. Red dots indicate the maximum intensity points; the red dotted line shows the leaf surface approximation that is the “leaf surface mask.” **c** The 3D surface plot of the “leaf surface mask.” **d** The virtual cut reflecting the cellular structure of the epidermal layer. **e** The segmented image on which a unique index marks each epidermal cell. Different colors correspond to different indices. **f-i** Correction of the “leaf surface mask” image for the pubescent leaf of wheat cv. Saratovskaya 29 for trichomes removing. **f** Initial 2D image with virtual cut carries trichomes complicating segmentation. **g** The “leaf surface mask” image taking into account the trichomes. **h** The “leaf surface mask” image with trichomes removed by mathematical morphology operations. **i** Resulting 2D image with virtual cut free of trichomes
- Segmentation by applying the Morphological Segmentation plugin [20, 26] for 2D/3D images containing information about the location of cell walls and nuclei. This plugin implements the algorithm of morphological segmentation through the watershed algorithm with markers [27], and can process both border images (cell walls) and object images (cell nuclei). Regions of cells can be isolated directly from the 3D image or the 2D image reconstructed before (Fig. 1a-e).
- Manual correction of defects for the segmented regions of cells (combining and removing of incorrectly segmented fragments). Automatic correction of defects for the segmented regions of nuclei (if the nucleus segment turned out to be fragmented and the boundary between the fragments has a width of one pixel). In addition, cells and nuclei can be grouped. For example, cells of the same type, or cells forming the same longitudinal cell row.
- Calculation of volumes for cells and nuclei based on the number of pixels in the corresponding region and meta-information from the lsm-file. If two segmented images are obtained, then the indices of cells and nuclei can be related to each other based on the relative location of the segmented regions.

The main functions of the plugin include:

- Formation and slice-by-slice visualization of 3D images in the form of separate color channels. Also the construction of a new image, which is the result of pixel linear operations with two (or more) channels. In some cases, the application of the segmentation algorithm for such a new image may result in fewer errors.
- Image quality improvement including the elimination of shifts and overlaps of the frames, and application of the anisotropic diffusion filter [23–25] in order to smooth and eliminate noise in the image, while maintaining the boundaries of cells and nuclei. The application of the filter significantly improves the quality of cell segmentation performed at the next processing step.
- Formation of a virtual cut reflecting the cellular structure of the epidermal layer (more details are below and in Fig. 1).

### Formation of a virtual cut reflecting the cellular structure of the epidermal layer

For the 3D images of cereal leaves, the following critical features were caught. Firstly, the outer part of the cell wall of the epidermal cells has a higher signal intensity than the lower one. Secondly, trichomes of pubescent wheat varieties rise above the epidermal layer creating a shadow, and their cell wall has a more intense signal than other epidermal cells. In connection with these features, segmentation of cells in the form of three-dimensional objects is often impossible. Therefore, the plugin has implemented a function forming a 2D image with a virtual cut reflecting the cellular structure of the epidermal layer. This algorithm is based on the assumption that the maximum of fluorescent signal intensity is concentrated on the outer part of the cell wall for the epidermal cells (Fig. 1a-e). In this case, if we make a virtual puncture through a stack of slices at each point of the XY-plane (Fig. 1a) and observe the change in the intensity of this signal along the Z-axis, then intensity maxima will be observed at points lying on the leaf surface (Fig. 1b). The set of these maximum points form a “leaf surface mask” (Fig. 1c). The intensity values at points lying inside the leaf and removed from the “leaf surface mask” for a fixed distance forms a 2D image with a virtual cut reflecting the cellular structure of the epidermal layer (Fig. 1d). Segmentation of such 2D images contains much fewer errors than segmentation of 3D image for the same leaf fragment. In addition, it requires less time and computing resources and in some cases is more convenient for further analysis (Fig. 1e). The “leaf surface mask” can be additionally edited with the help of filters (such as mean or median filters) and grayscale mathematical morphology [28] operations, in particular, to remove external outgrowths of trichomes, if they do not participate in further analysis (Fig. 1 f-i). In the case of 2D segmentation, to calculate the correspondence between cells and nuclei, the plugin uses a parameter of the epidermal layer depth and approximates each cell with a cylinder with base corresponding to the 2D projection of the cell.

To implement the plugin, we used the ImageJ platform [29] which has an open source code in the Java language and its plugins are used for a wide range of tasks in biological image analysis and processing. The complete class diagram is shown on the Figure S1 (Additional file 2). A detailed manual containing all the functions of LSM-W^2^ is given in Additional file 3.

### Further possibilities of data analysis for LSM-W^2^ outputs

2D images of virtual cuts reflecting the cellular structure of the epidermal layer constructed with LSM-W^2^ from confocal 3D images are suitable for further manual analysis and allow an expert marking with other tools. For example, we used an ImageJ plugin CellCounter [30] in our work [6] for marking the aberrations of stomatal morphogenesis in the epidermis of boot leaves of wheat in response to cold stress. In addition, such images can be analyzed further using other programs, such as CellSeT [14], implementing alternative cell segmentation algorithms.

Topology and geometry are two critical aspects that should be considered when clarifying the mechanisms that determine the development of epidermal leaf tissue. Geometric characteristics include the shape and size of the cells, while topological characteristics refer to the connectivity of cells within the tissue, and can be characterized, for example, by the number of neighbors of each cell. Segmented images extracted with LSM-W^2^ from lsm-files consist of cell regions marked with unique indices can and serve as a rich source of data on the morphological properties of epidermal cells. Marked images can serve as part of a plant phenotype study produced by a high-level general-purpose programming language such as Mathematica, MathLab, and Python that have rich libraries of functions for image analysis whereby morphometric properties for a large number of cells can be extracted. These data can serve as the basis for constructing spatial diagrams and studying the spatial distribution of the morphological properties of cells through a leaf.

The results of measurements and comparisons of the volume of cells and nuclei extracted by LSM-W^2^ in the form of csv-files can be further processed using multidimensional data analysis methods.

## Results and discussion

In this section, we discuss four case studies showing the applying of the proposed plugin for solving biological tasks.

### Case study 1. Defining parameters of a jigsaw-puzzle pattern for maize leaf epidermal cells

In vascular plants, leaf epidermal pavement cells often have wavy contours due to the formation of lobes by anticlinal cell walls. Currently, the mechanisms of jigsaw-puzzle pattern formation in the process of plant development are issues for consideration. Image analysis methods play a significant role including using mathematical modeling to test hypotheses about possible details of these mechanisms [31]. Molecular signals, whose targets are the cytoskeleton and cell wall, are actively studied on Arabidopsis and maize. Thus, the review [32] deals with studies concerning mechanisms of puzzle structure formation in the epidermis of Arabidopsis. It has been shown that mutations affecting microtubules on microtubule-associated processes and/or actin cytoskeleton result in reduced lobing and, accordingly, the formation of polyhedral cells instead of wild-type cells. Higaki and co-authors proposed a model of epidermal patterning for the Arabidopsis leaf [33]. The degree of expression for the jigsaw-puzzle structure was estimated as the ratio of the perimeter of a young cell to the perimeter of a more mature cell and was verified by images. Although recently some studies were performed on the development of puzzle-shaped cells, more measurements on the growth of the anticlinal walls during lobe formation are needed to clarify the issue [34].

In this case study, we show how the proposed plugin can be used to obtain data on the cellular properties for the maize leaf epidermis in the form of a map of segmented cells. Further, the characteristics of the epidermal cells from the point of view of their “lobeyness” were obtained.

**Fig. 2:**
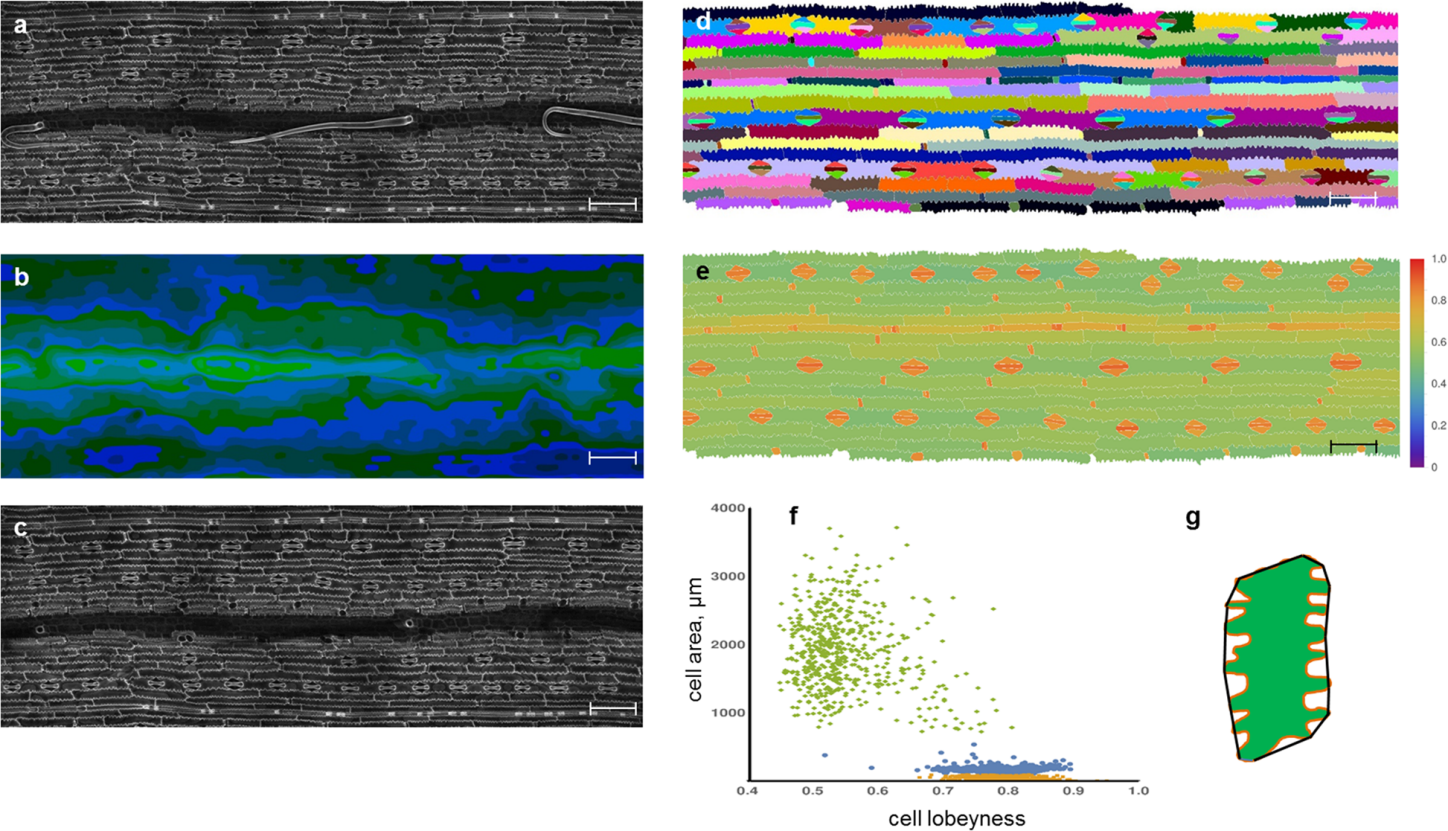
Defining parameters of the epidermal jigsaw-puzzle cell pattern for mature maize leaf. **a** 2D image of the virtual cut reflecting the cellular structure of the epidermal layer with trichomes. **b** Heat map of the “leaf surface mask” with different colors corresponding to different values of z-slice number. The “leaf surface mask” was corrected in order to remove trichomes from the image A. **c** 2D image of the virtual cut reflecting the cellular structure of the epidermal layer without trichomes. **d** Watershed cell segmentation with manual correction for the maize leaf fragment. **e** Heat map marking of cells based on their “lobeyness”. Scale bars for A-E: 100 *μ*m. **f** Scatter plot of cells’ area vs. cells’ “lobeyness” with Canberra distance function for three clusters: pavement cells (green), stomata (blue), and trichomes and other small cells (orange). The maize leaf fragment containing 1325 cells was used for the analysis. **g** Explanation of the term “lobeyness”, which is the ratio of the cell’s perimeter (orange) to the cell’s convex hull perimeter (black)

A fragment of the tenth leaf of maize (*Zea mays* L.) inbred line 611 originated from cv. Mo17 (kindly provided by Pavel Panikhin from Institute of Molecular and Cellular Biology SB RAS, Novosibirsk) was stained and scanned according to the protocol (see Methods in Additional file 1). As a result, a 3D image for a 2334.72 × 778.24×42 *μ*m leaf fragment was obtained. Using the LSM-W^2^ plugin, the fragments were combined into a general 3D image, and a 2D image of the virtual cut reflecting the cellular structure of the epidermal layer was obtained (Fig. 2a). The “leaf surface mask” was corrected in order to remove trichomes from the image (Fig. 2b), improved 2D image (Fig. 2c) further was segmented using the Morphological Segmentation plugin integrated into the LSM-W^2^ (Fig. 2d). The segmented image allows quantifying the parameters of the cellular pattern. In this case study, we estimated the degree of waviness for pavement epidermal cells. To perform this task, we used the parameter “lobeyness” which is the ratio of the cell convex hull perimeter to the cell perimeter [4]. “Lobeyness” gives a measure of how lobed the cell is. In the Fig. 2e, cells consisting the maize leaf epidermis are marked with different colors, depending on the value of their “lobeyness”. Pavement cells are marked with values close to 0.6 since their cell wall is the waviest. The undulating structure of the puzzle becomes less specific above the vessels. Cells of the stomata, trichomes, and other small cells are well distinguished from pavement cells by the value of “lobeyness”. Estimating the relationship between “lobeyness” and cell area (Fig. 2g), we can define the following three clusters: pavement cells, stomata (guard and subsidiary) cells, and trichomes. The analysis is performed using the built-in functions of the Matematica 10 package.

In addition to the above case study, the plugin can be useful for estimating the area of outgrowths in mutant forms and wild-type. With the help of a plugin, it is also possible to estimate the area of intercellular contacts, which is essential for studying signaling and intercellular adhesion.

### Case study 2. Analysis of the pavement cells morphological parameters for the mature wheat leaf under control and water deficit conditions

Water deficiency is one of the most important factors that reduce the yield of cereal crops. In this regard, studies of the tolerance of agricultural plants to drought are of great importance [35, 36]. It was shown that a wide range of plant species reveal the changes in the size and density of stomata and pavement epidermal cells during drought stress [37–39]. Previously, using LHDetect leaf fold image processing software [40], we found that there is an increase in the number of trichomes on a wheat leaf with a decrease in their length in response to drought stress [41]. However, this method cannot identify the differences for glabrous wheat varieties that carry the recessive phenotype of pubescence (such as cv. Rodina). The cellular pattern of the leaf epidermis of such varieties contains a significant number of cells morphologically corresponding to short trichomes. According to microscopic data, these cells have a small basal part and a pronounced cone-shaped outgrowth with a thickened cell wall, a height of 2-5 *μ*m. LHDetect2 allows analyzing trichomes on a leaf fold, but it cannot recognize trichomes of the extremely small size. While the LSM-W^2^ plugin allows analyzing the leaf surface, therefore, main epidermal cells may also be involved in the analysis.

The main epidermal cells protect the internal leaf tissues from the external environment and make a decisive contribution to the biomechanics of leaf growth. Therefore, changes in the morphological parameters of the main epidermal cells can act as an indicator of stress-induced changes in plant growth and morphogenesis. To understand the mechanisms of epidermal cellular structure formation, we need to identify the relative location and sizes of the main epidermal cells. These parameters can be obtained using the LSM-W^2^ plugin.

As a case study, we propose an analysis of the main epidermal cells morphological parameters in the leaves of the wheat cv. Rodina in favorable and water deficiency conditions. Plants were grown in of Mitcherlich containers, filled with 4 kg of expanded clay balls. The favorable conditions corresponded to 60%, and the water deficit conditions corresponded to 30% of the water from the total moisture capacity of the substrate, which was determined as described in the manual [42]. The photoperiodic day/night cycle was 186 hours; the temperature condition was maintained at 14-16 ^∘^C at night and 20-23 ^∘^C during the day. A sodium-vapor lamp provided lighting; illumination of plants was carried out as 15−20·10^3^ lx. A 20 mm length fragments from the central part of boot leaves of wheat plants at the heading stage were used for comparative analysis.

Using the LSM-W^2^ plugin, we converted 3D-images into 2D-images and segmented cells (projections) and the nuclei (volumes). Using the function embedded in the plugin, cells and nuclei were mapped, and data on its sizes were saved as a csv-table. A total number of 874 main epidermal cells of control plants and 519 cells of plants subjected to stress were measured. Using the built-in functions of the Mathematica 10 package (ComponentMeasurements function) for segmented images, the morphological parameters of the cells were determined (length, width, elongation computed as 1-widthlength, and circularity computed as the ratio of equivalent disk perimeter to the perimeter length).

**Fig. 3:**
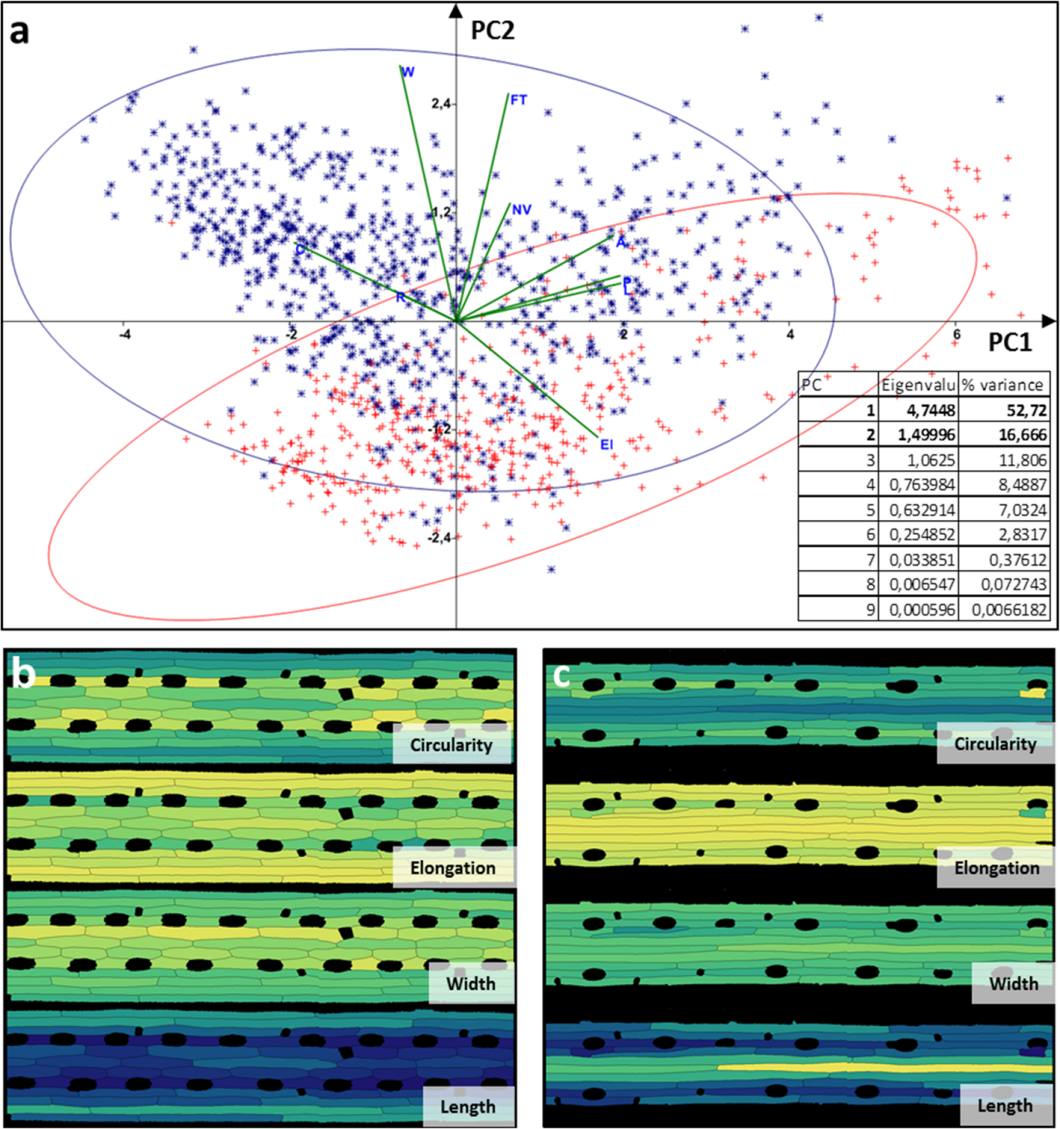
Analysis of the pavement cells morphological parameters for wheat cv. Rodina under control and water deficit conditions. **a** Principal component analysis (PCA) of morphological parameters (area (A), perimeter (P), length (L), width (W), elongation (El), circularity (C), rectangularity (R) of cell’s projection, volume of cell’s nuclear (NV), and type of longitudinal cell row (FT)) for the pavement cells under control (blue star), and water deficit (red cross) conditions; ellipses contain 95% of the sample elements. **b-c** Heat map marking for a fragment of the leaf epidermal pattern between two adjacent vessels based on morphological parameters of cells (length, width, elongation, and circularity) for control (B) and water deficit conditions (C). 874 cells were segmented for control, and 519 cells were segmented for water deficit conditions. The change in color from blue to yellow corresponds to a change in the corresponding parameter from the minimum to the maximum values

Principal component analysis (PCA) was applied to identify what morphological parameters of cells are most significant for detecting the effect of stress. The following values were used in PCA: (i) morphological parameters for the pavement cells evaluated by 2D cell-segmented image (area, perimeter, largest axis of the best-fit ellipse (length), smallest axis of the best-fit ellipse (width), elongation (computed as 1-widthheight), circularity (computed as ratio of equivalent disk perimeter to the perimeter length), rectangularity (computed as fraction of pixels within the minimal bounding box); (ii) volume of cell nucleus; and (iii) type of the cell longitudinal row (determined by an expert). We found that cell length and perimeter make the most significant contribution to PC1 (Fig. 3a). Also, we showed that in the conditions of water deficiency the morphological parameters of the main cells of the epidermis vary significantly (blue stars and red crosses on Fig. 3a). This difference is well noticeable in the diagrams constructed for leaf fragments between two adjacent vessels. The diagrams show the spatial distribution for some parameters used in the PCA (Fig. 3b-c). As a result, it was found that under the conditions of water deficiency, the parameters of the pavement epidermal cells change significantly. For a visual representation of the difference between control and water-deficit conditions, heat maps representing the spatial distribution of four parameters on the leaf fragments between two vessels were constructed (Fig. 3b-c). In addition to changes in morphological parameters of the pavement cells, the overall leaf width also decreased under the conditions of water deficit.

### Case study 3. Determination of longitudinal cell rows initiation in the wheat leaf growth zone

To clarify the mechanisms for the leaf epidermal pattern formation of cereals, it is necessary to study the mutual arrangement of cells in the growth zone. Morphogenesis of cereals leaf epidermis consists of several stages differing by the morphological features of the cells. In the meristem zone cells have almost round shape, then in the zone symmetrical divisions cells begin to elongate and organize longitudinal cell rows where division in the direction perpendicular to the leaf growth axis are allowed, then in the zone of asymmetric divisions specializing longitudinal cell rows is located. Definition of boundaries for the zone of longitudinal cell rows determinism is a topic for this case study. The aim was to classify cells according to their morphology that may predict their future specialization.

**Fig. 4:**
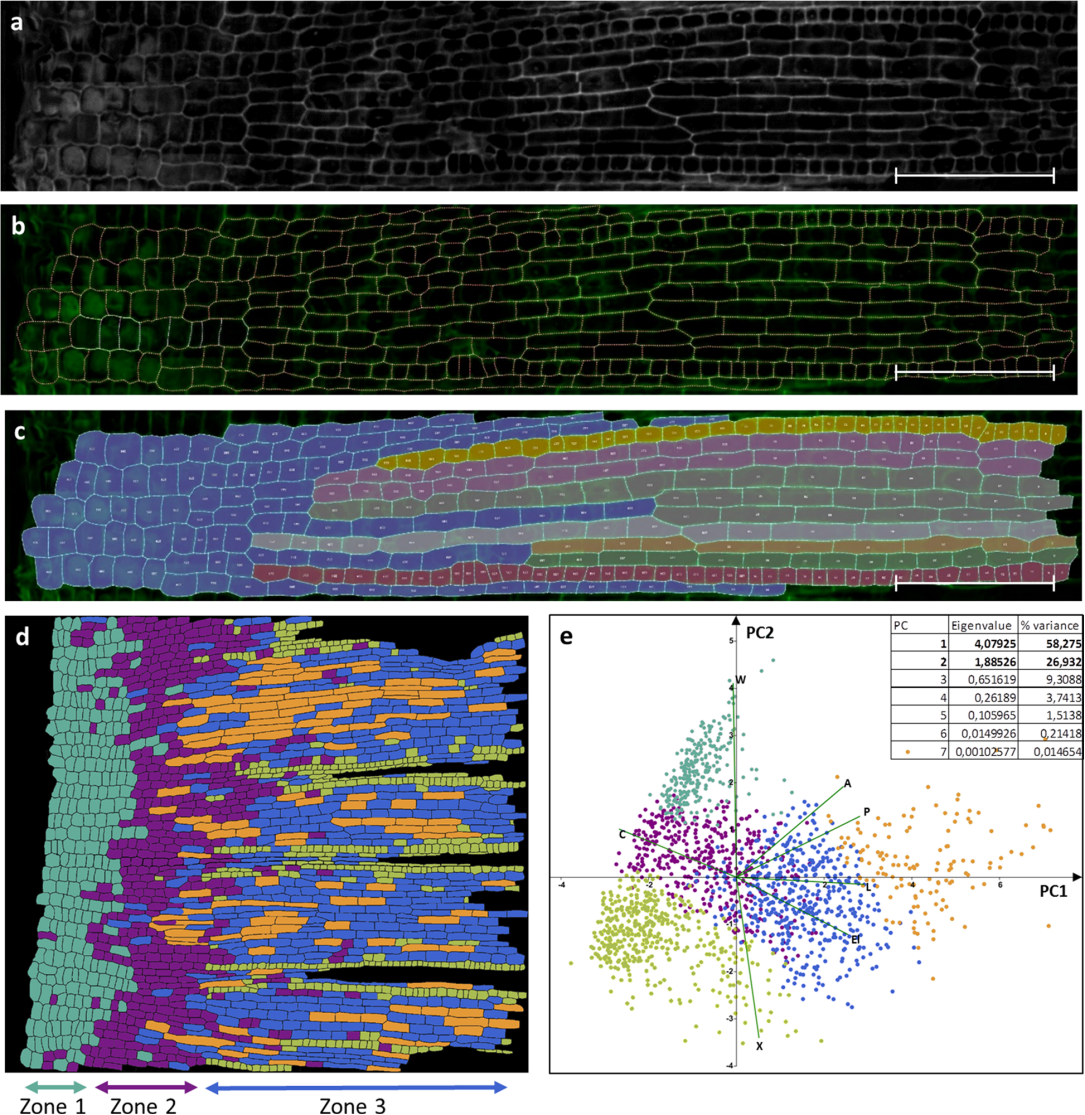
Epidermal cell pattern in the growth zone of the wheat leaf. **a**–**c** The processing steps are demonstrated on a small part of the image. **a** The virtual cut reflecting the cellular structure of the epidermal layer. **b** Segmentation of the cell walls performed using a program CellSeT [14]. **c** Manual marking of cells types based on longitudinal cell rows emergence. Scale bars for A-C: 100 *μ*m. **d** The diagram of spatial distribution for cell types on the whole fragment (1721 cells). Cells were clustered by K-means for five clusters based on x coordinate of the centroid, area, perimeter, length, width, elongation, circularity. **e** Principal component analysis of morphological parameters for the epidermal cells in the growing zone (area (A), perimeter (P), length (L), width (W), elongation (El), circularity (C), rectangularity (R) of cell’s projection). Colors of points indicate cell type and correspond to the diagram (D)

In this case, we studied changes in the epidermal cellular pattern in the growth zone for the epidermis of the fifth leaf of wheat cv. Chinese spring (from the genetic collection of the Institute of Cytology and Genetics SB RAS kindly provided by Tatyana Pshenichnikova). Samples of leaves fragments were stained with DAPI and PI according to the standard procedure described in Additional file 1. The resulting two-channel 3D image containing information on the position of the cell walls and nuclei was processed according to the procedure described above. Figure 4a-c shows the steps of image processing. The virtual cut reflecting the cellular structure of the epidermal layer was obtained with the plugin LSM-W^2^ (Fig. 4a). By this image, segmentation of the cell walls was performed using a program *CellSeT* [14]. As a result, the vertex model of the epidermal cell structure was constructed. Figure 4c shows an example of manual marking of longitudinal cell rows. According to this model, the following parameters were measured: cell length, number of neighbors, area. The dependence of these parameters on the spatial position of the cell inside the pattern and the current stage of differentiation was estimated. Morphological properties of 1721 cells were analyzed. Cells were clustered by K-means for five clusters based on morphological properties of its shapes. The diagram (Fig. 4 d) shows the spatial distribution for cell types on the whole fragment. Cluster analysis made it possible to identify (i) a zone composed of round anisotropically dividing cells, (ii) a transition zone, and (iii) a zone where the determination of longitudinal cell rows begins. PCA reveals that cell length and perimeter make the major contribution in PC1 (Fig. 4 e). In the first zone, the cells have almost the same values of the area, in the second zone the values of the parameter were divided into a group of large cells, and a group of small ones, and in the third zone longitudinal rows of specialized cells were isolated. The average area of the cell in the tissue does not depend on the zone and remains constant. Data on the distribution of cell sizes along the leaf are useful for verifying cell-oriented growth models for linear leaves [43].

### Case study 4. Specification of stomata guard mother cells in the wheat leaf growth zone

The mechanisms of the specialized epidermal structures formation require a comprehensive consideration because of their fundamental role for plant development. The vital nature of the processes occurring during the formation of stomata is emphasized by the interest of researchers [44, 45].

A distinctive feature of the epidermal pattern of cereals (in particular, wheat and maize) is the organization of cells in the form of longitudinal cell rows. In this pattern, certain longitudinal cell rows are predetermined for the formation of certain specialized cells, in particular trichomes and stomata. In the process of growth, the stages of morphogenesis occur successively along the leaf growth axis, and this allows one to observe several stages simultaneously. Thanks to this, the epidermis of a cereal leaf is a convenient object for studying morphogenesis. For the formation of stomata, at the distal end of the meristemoid mother cell, a small initial separates by asymmetric division and subsequently forms a specialized cellular structure of the stomata consisting of several cells.

**Fig. 5:**
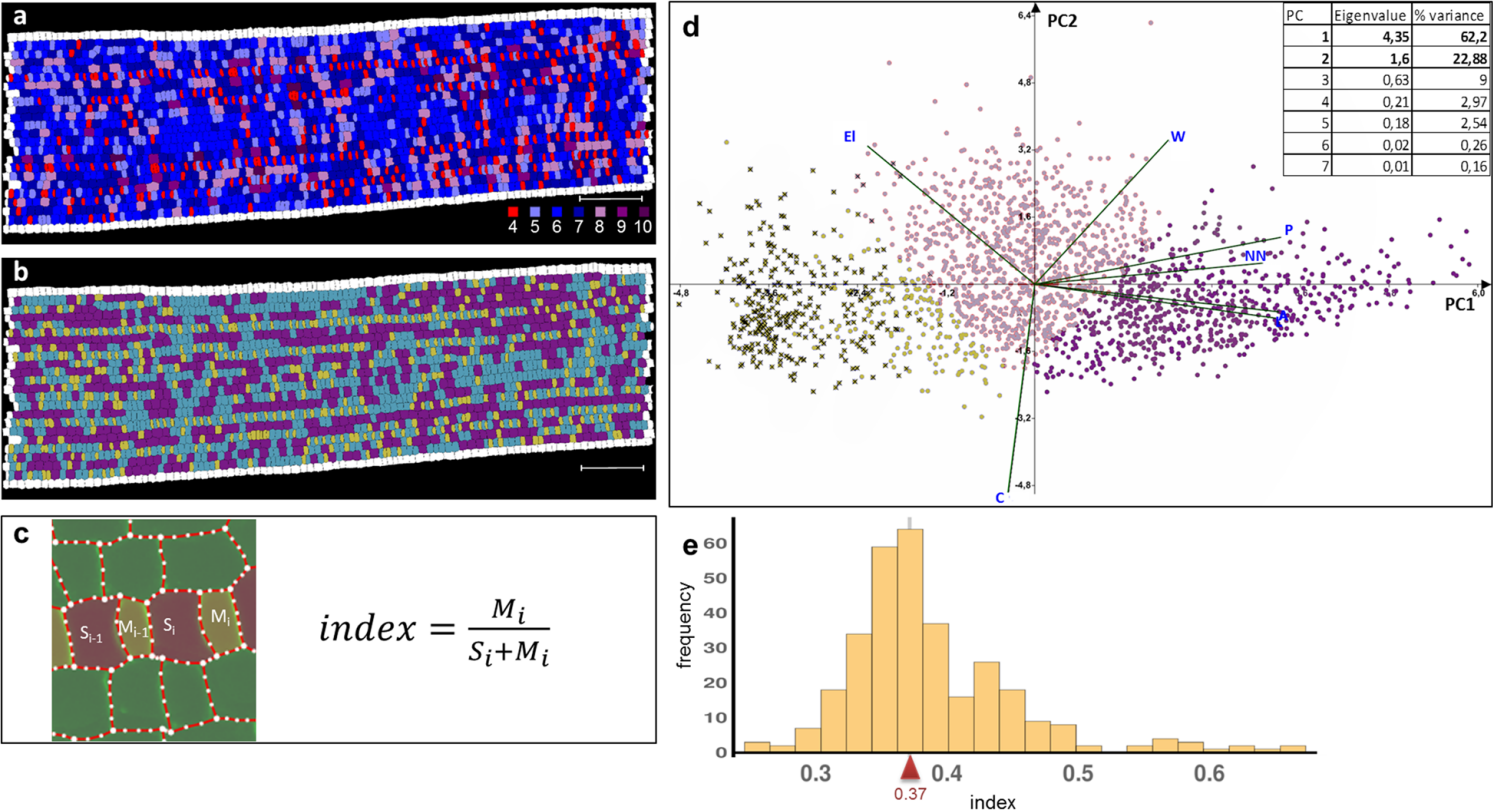
Specification of stomata guard mother cells in the wheat leaf growth zone. **a** Diagram of the neighbor count for each cell of analyzed leaf fragment. The marginal cells were considered as neighbors but did not participate in the further analysis. **b** Diagram of K-means clustering of cells on the basis of their neighbor count and morphometric properties (area, perimeter, length, width, circularity, elongation) for three clusters. Scale bars for A and B: 100 *μ*m. **c** Scheme of manual cell classification: pavement cells (green), stomata guard mother cells (yellow), pavement cells in stomata cell row (red) and formula for cell size index calculation, where M is area of guard mother cell, and S is area of a sister cell for guard mother cell which will not form stomata. **d** Principal components analysis for morphometric properties of cells. Point colors on the scatter plot corresponds to cell colors on diagram (E). Crosses indicate cells that have four neighbors. **e** Histogram of cell size index, the red arrow indicates the median of the distribution

The first stage in the stomata morphogenesis forming a guard mother cell is critical for the formation of a stomata pattern for the whole leaf. It is essential that stomata be smaller than the pavement cells in adjacent rows, which will continue actively growing in length when the stomata will differentiate. Therefore, the guard mother cell forms a specific structure (Fig. 5c), in which the cell wall between it and the cell from neighboring row has a small length, and the walls facing the inside of the same row has a curved shape and consequently is longer. Before the next division, the guard mother cell induces asymmetric divisions in abutting longitudinal cell rows. These divisions produce subsidiary cells, which also will form elements of the stomata. Since subsidiary cells are recruited from the neighboring rows, it is necessary that the boundary of guard mother cell is not located at the junction of two cells from a neighboring row. For this reason, the guard mother cell usually has four neighbors. At later stages of morphogenesis, the guard mother cell symmetrically divides and forms two guard cells. Thus, properly formed wheat stomata consist of four cells [3, 46–50].

A fragment from the wheat leaf growth zone (cv. Rodina) at the stage of asymmetric divisions was scanned. The resulting 3D image was processed using the LSM-W^2^ plugin. Since guard mother cell usually has four neighbors, we can automatically select this type of cells (Fig. 5a). Selection of clusters according to the neighbor count and morphometric properties (area, perimeter, length, width, circularity, elongation) makes it possible to state that the mother guard cells are well distinguishable from other cells according to these parameters (Fig. 5b). Therefore, for the automatic analysis of precursors of specialized epidermal structures and the cell longitudinal row formation, this method of cell selection is suitable. PCA shows that in PC1 the following parameters (perimeter, neighbor count, area, and cell length) make a major contribution, while in PC2 circularity with negative correlation makes a major contribution. We introduced a cell size index, which is the ratio of the selected cell area and the sum of the areas of the cell and its left neighbor. These cells are direct descendants of one cell. Figure 5e shows the histogram of cell size index for the cells with four neighbors. The median for this distribution is 0.37. Cells with index values greater than 0.5 are probably trichome precursors that are less ordered and have a smaller difference between sister cells’ areas. Therefore, using the LSM-W^2^ plugin, it is possible to automatically isolate and measure the guard cell and thus trace the further fate of the development of the epidermal patterns for the cereal leaf, which is important for the study of morphogenesis.

## Conclusion

In modern biological science, interdisciplinary system approaches play an important role. These approaches allow accessing the mechanics of genotype-phenotype interactions, evolution, and development. In many issues of biology, the limiting factor is a not enough high-performance description of the phenotype at the cellular and tissue levels.

Microscopic images are an important source of information on morphometric characteristics of the cells and the statistical analysis of cellular tissue architecture. High-resolution 3D confocal images allow extracting quantitative characteristics describing the cell structure of leaf tissues. However, to obtain a large number of statistical data methods of high throughput computer-based image segmentation should be used.

In this particular work, we performed a new high throughput workflow for detection of structural properties of leaf epidermis from 3D images obtained from confocal laser scanning microscopy. Characteristics of the cell structure and patterns extracted from these images further will act as a basis for the development and verification of spatial models of plant tissues formation mechanisms accounting for structural features of cereal leaves. The central element of the workflow is a new ImageJ-plugin, which processes raw and large-scale lsm-scans and extracts virtual cuts reflecting cellular structure for tissue of interest allowing to obtain morphometric data both by itself and/or by using additional software or other ImageJ-plugins.

We have performed several case studies to show the workflow efficiency and flexibility. We obtain statistical characteristics of the cellular pattern for leaf epidermis of two spring varieties of bread wheat characterized by different morphological features of epidermal cells and grown in water deficit and favorable conditions. Obtained data provide material for formulation hypotheses and modeling variation between phenotypes during variety-specific leaf growth. We carried out a classification of the cells of the growing leaf zone according to morphological characteristics. We have shown the ability to stably distinguish the various zones along the developing leaf using a number of predictors and to detect events of asymmetric division and its parameters. Besides, we defined the parameters of the epidermal jigsaw-puzzle cell pattern for mature maize leaf. The results of this analysis showed the possibility of rapid adaptation of the method to a wide range of plant species, primarily cereals. The results demonstrate that the proposed method is rapid, adequately assesses structure characteristics of leaf epidermis, and the data obtained by this method significantly correlate with manual measurement of cell lengths. Thus, the proposed method is efficient for high-throughput analysis of cell architecture of leaf epidermis in cereal genetic experiments and selection. Our technique allows one to study morphogenesis using inexpensive and rapid sample preparation and does not require the use of transgenesis and reporter genetic structures, expanding the range of species and varieties to study.

## Availability and requirements

**Project name:** LSM-W ^2^

**Project home page:**https://imagej.net/LSM_Worker, https://github.com/JmanJ/LSM_Worker and https://pixie.bionet.nsc.ru/LSM_WORKER/

**Operating system(s):** Windows, Mac OS, Linux

**Programming language:** Java

**Other requirements:** ImageJ launcher (Fiji)

**License:** FreeBSD

**Any restrictions to use by non-academics:** no

## Additional files

Additional file 1 Staining and microscopy protocol. (DOCX 15 kb)

Additional file 2 LSM-W^2^ user manual. (DOCX 1266 kb)

Additional file 3 LSM-W^2^ class diagram. (JPG 401 kb)

## Abbreviations

CW: Calcofluor-white
DAPI: 4’,6-diamidino-2-phenylindole
LSM: Laser scanning microscopy
PC: Principal component
PCA: Principal components analysis
PI: Propidium iodide

## References

[1] Meijering E (2012). Cell segmentation: 50 years down the road [life sciences]. IEEE Signal Proc Mag.

[2] Robinson DO, Roeder AH (2015). Themes and variations in cell type patterning in the plant epidermis. Curr Opin Genet Dev.

[3] Carter R, Sánchez-Corrales YE, Hartley M, Grieneisen VA, Marée AF (2017). Pavement cells and the topology puzzle. Development.

[4] Sapala A, Runions A, Routier-Kierzkowska A-L, Gupta MD, Hong L, Hofhuis H, Verger S, Mosca G, Li C-B, Hay A (2018). Why plants make puzzle cells, and how their shape emerges. ELife.

[5] Andriankaja M, Dhondt S, De Bodt S, Vanhaeren H, Coppens F, De Milde L, Mühlenbock P, Skirycz A, Gonzalez N, Beemster GT (2012). Exit from proliferation during leaf development in arabidopsis thaliana: a not-so-gradual process. Dev Cell.

[6] Zubairova U, Doroshkov A (2018). Wheat leaf epidermal pattern as a model for studying the influence of stress conditions on morphogenesis. Vavilovskii Zh Genetiki i Selektsii =Vavilov J Genet Breed.

[7] Lobet G. The Plant Image Analysis Software Database. http://www.plant-image-analysis.org. Accessed 30 Oct 2018.

[8] Lobet G, Draye X, Périlleux C (2013). An online database for plant image analysis software tools. Plant Methods.

[9] Lobet G (2017). Image analysis in plant sciences: publish then perish. Trends Plant Sci.

[10] de Reuille PB, Routier-Kierzkowska A-L, Kierzkowski D, Bassel GW, Schüpbach T, Tauriello G, Bajpai N, Strauss S, Weber A, Kiss A (2015). Morphographx: a platform for quantifying morphogenesis in 4d. Elife.

[11] Mosaliganti KR, Noche RR, Xiong F, Swinburne IA, Megason SG (2012). Acme: automated cell morphology extractor for comprehensive reconstruction of cell membranes. PLoS Comput Biol.

[12] Schmidt T, Pasternak T, Liu K, Blein T, Aubry-Hivet D, Dovzhenko A, Duerr J, Teale W, Ditengou FA, Burkhardt H (2014). The irocs toolbox–3 d analysis of the plant root apical meristem at cellular resolution. Plant J.

[13] Blein T, Duerr J, Pasternak T, Haser T, Falk T, Liu K, Ditengou FA, Ronneberger O, Palme K. Light dynamically regulates growth rate and cellular organisation of the arabidopsis root meristem. BioRxiv. 2018:353987. 10.1101/353987. Accessed 30 Oct 2018.

[14] Pound MP, French AP, Wells DM, Bennett MJ, Pridmore TP (2012). Cellset: novel software to extract and analyze structured networks of plant cells from confocal images. Plant Cell.

[15] Ivanov OV, Pyatnitskiy AM, Ignatov MS, Maslova EV (2013). Areoana analysis of moss leaf cell structure of two cyrtomnium species (mniaceae, bryophyta). Arctoa.

[16] Schindelin J, Arganda-Carreras I, Frise E, Kaynig V, Longair M, Pietzsch T, Preibisch S, Rueden C, Saalfeld S, Schmid B (2012). Fiji: an open-source platform for biological-image analysis. Nat Methods.

[17] Pirrotte P, Mutterer J. LSM Toolbox Plugin. http://imagejdocu.tudor.lu/doku.php?id=plugin:inputoutput:lsmtoolbox:start. Accessed 30 Oct 2018.

[18] Preibisch S, Saalfeld S, Tomancak P (2009). Globally optimal stitching of tiled 3d microscopic image acquisitions. Bioinformatics.

[19] Möller B, Poeschl Y, Plötner R, Bürstenbinder K (2017). Pacequant: a tool for high-throughput quantification of pavement cell shape characteristics. Plant Physiol.

[20] Legland D, Arganda-Carreras I, Andrey P (2016). Morpholibj: integrated library and plugins for mathematical morphology with imagej. Bioinformatics.

[21] Wu T-C, Belteton S, Pack J, Szymanski DB, Umulis D (2016). Lobefinder: a convex hull-based method for quantitative boundary analyses of lobed plant cells. Plant Physiol.

[22] Jonsson H. Costanza. http://www.plant-image-analysis.org/software/costanza. Accessed 30 Oct 2018.

[23] Pilny V, Janacek J. Anisotropic Diffusion Plugin. https://imagej.nih.gov/ij/plugins/anisotropic-diffusion-2d.html. Accessed 30 Oct 2018.

[24] Perona P, Malik J (1990). Scale-space and edge detection using anisotropic diffusion. IEEE Trans Pattern Anal Mach Intell.

[25] Tschumperle D, Deriche R (2005). Vector-valued image regularization with pdes: A common framework for different applications. IEEE Trans Pattern Anal Mach Intell.

[26] Arganda-Carreras I, Legland D. Morphological Segmentation Plugin. https://imagej.net/Morphological_Segmentation. Accessed 30 Oct 2018.

[27] Vincent L, Soille P (1991). Watersheds in digital spaces: an efficient algorithm based on immersion simulations. IEEE Trans Pattern Anal Mach Intell.

[28] Serra J, Soille P. Mathematical Morphology and Its Applications to Image Processing, vol 2.Springer; 2012. 10.1007/978-94-011-1040-2.

[29] Abramoff MD, Magalhaes PJ, Ram SJ (2004). Image processing with imagej. Biophoton Int.

[30] Vos KD. Cell Counter Plugin. https://imagej.nih.gov/ij/plugins/cell-counter.html. Accessed 30 Oct 2018.

[31] Majda M, Grones P, Sintorn I-M, Vain T, Milani P, Krupinski P, Zagórska-Marek B, Viotti C, Jönsson H, Mellerowicz EJ (2017). Mechanochemical polarization of contiguous cell walls shapes plant pavement cells. Dev Cell.

[32] Kotzer A, Wasteneys G (2006). Mechanisms behind the puzzle: microtubule–microfilament cross-talk in pavement cell formation. Botany.

[33] Higaki T, Kutsuna N, Akita K, Takigawa-Imamura H, Yoshimura K, Miura T (2016). A theoretical model of jigsaw-puzzle pattern formation by plant leaf epidermal cells. PLoS Comput Biol.

[34] Borowska-Wykret D, Kwiatkowska D. Folding, Wrinkling, and Buckling in Plant Cell Walls In: Geitmann A, Gril J, editors. Plant Biomechanics. Springer: 2018. p. 209–233. 10.1007/978-3-319-79099-2_10.

[35] Hughes J, Hepworth C, Dutton C, Dunn JA, Hunt L, Stephens J, Cameron D, Waugh R, Gray JE (2017). Reducing stomatal density in barley improves drought tolerance without impacting on yield. Plant Physiol.

[36] Caine RS, Yin X, Sloan J, Harrison EL, Mohammed U, Fulton T, Biswal AK, Dionora J, Chater CC, Coe RA, et al.Rice with reduced stomatal density conserves water and has improved drought tolerance under future climate conditions. New Phytol. 2018; 0. 10.1111/nph.15344. 10.1111/nph.15344 PMC6492113 30043395

[37] Bosabalidis AM, Kofidis G (2002). Comparative effects of drought stress on leaf anatomy of two olive cultivars. Plant Sci.

[38] Makbul S, Guler N, Durmus N, Guven S (2011). Changes in anatomical and physiological parameters of soybean under drought stress. Turk J Bot.

[39] Esmaeilpour A, Van Labeke M-C, Samson R, Boeckx P, Van Damme P (2016). Variation in biochemical characteristics, water status, stomata features, leaf carbon isotope composition and its relationship to water use efficiency in pistachio (*Pistacia vera* l.) cultivars under drought stress condition. Sci Hortic.

[40] Genaev MA, Doroshkov AV, Pshenichnikova TA, Kolchanov NA, Afonnikov DA (2012). Extraction of quantitative characteristics describing wheat leaf pubescence with a novel image-processing technique. Planta.

[41] Doroshkov A, Pshenichnikova T, Afonnikov D (2011). Morphological characterization and inheritance of leaf hairiness in wheat (*Triticum aestivum* l.) as analyzed by computer-aided phenotyping. Russ J Genet.

[42] Zhurbitzkiy ZI (1968). Theory and Practice of Vegetation Method.

[43] Zubairova U, Nikolaev S, Penenko A, Podkolodnyy N, Golushko S, Afonnikov D, Kolchanov N (2016). Mechanical behavior of cells within a cell-based model of wheat leaf growth. Frontiers Plant Sci.

[44] Geisler M, Nadeau J, Sack FD (2000). Oriented asymmetric divisions that generate the stomatal spacing pattern in arabidopsis are disrupted by the too many mouths mutation. Plant Cell.

[45] Bergmann DC (2004). Integrating signals in stomatal development. Curr Opin Plant Biol.

[46] Liu T, Ohashi-Ito K, Bergmann DC (2009). Orthologs of arabidopsis thaliana stomatal bhlh genes and regulation of stomatal development in grasses. Development.

[47] Gallagher K, Smith LG (2000). Roles for polarity and nuclear determinants in specifying daughter cell fates after an asymmetric cell division in the maize leaf. Curr Biol.

[48] Peterson KM, Rychel AL, Torii KU (2010). Out of the mouths of plants: the molecular basis of the evolution and diversity of stomatal development. Plant Cell.

[49] Rudall PJ, Chen ED, Cullen E (2017). Evolution and development of monocot stomata. Am J Bot.

[50] Hepworth C, Caine RS, Harrison EL, Sloan J, Gray JE (2018). Stomatal development: focusing on the grasses. Curr Opin Plant Biol.

