# Supplementary material for: LSM-W2: laser scanning microscopy worker for wheat leaf surface morphology
Source: BMC Syst Biol. 2019 Mar 5;13(Suppl 1):22. doi: 10.1186/s12918-019-0689-8 (PMC6399813; doi:10.1186/s12918-019-0689-8)
Supplement: Supplementary file 1 — Staining and microscopy protocol. (DOCX 15 kb) [file 12918_2019_689_MOESM1_ESM.docx]

## **Additional file 1. Staining and microscopy protocol**

For successful segmentation, in the input images, the cell walls and nuclei of the epidermal cells of the leaf should be distinguishable, and the background signal should be as low as possible. This aim was achieved by staining samples of leaves fragments with a set of fluorescent dyes. Dyes 4',6-diamidino-2-phenylindole (DAPI, Sigma-Aldrich), Calcofluor-White (CW, Sigma-Aldrich) and Propidium Iodide (PI, Sigma-Aldrich) were used for staining. This set of dyes on a fixed material provides a stable to fading staining for nuclei and cell walls and makes it possible to distinguish deep-lying vessels. We used dyes according to the methods (Arumuganathan and Earle, 1991; Arumuganathan and Earle, 1991a; Kapuscinski, 1995; Harrington and Hageage, 2003) with changes. Staining and washing times were determined empirically. Freshly harvested fragments of the leaves were incubated in the ethanol: acetic acid (3:1) fixative solution for three or more hours. The staining was carried out in two stages with intermediate washing with neutral buffer (Phosphate Buffered Saline (PBS) Tabs pH 7.4, Thermo Fisher Scientific) according to one of the following protocols:

**Protocol 1 Staining of the mature leaves.**

- wash the samples with PBS without agitation for 15 minutes twice,
- place the samples in a PI solution (10 μg / ml in PBS),
- wash again with PBS twice for 15 minutes,
- stain the samples with DAPI 10 μg / ml solution for 30 minutes, and it is also possible to use the special mounting medium with DAPI,
- place the samples in a mounting medium with DABCO* (Sigma-Aidrich) on a glass slide and covered with coverglass.

**Protocol 2 Staining of leaf fragments from the growth zone.**

- wash the samples with PBS without agitation for 15 minutes twice,
- place the samples in a PI solution (10 μg / ml in PBS),
- wash again with PBS twice for 15 minutes,
- stain the samples with CW 10 μg / ml solution for 30 minutes,
- place the samples in a mounting medium with DABCO* (Sigma-Aidrich) on a glass slide and covered with coverglass.

Then all slides were examined under a microscope and scanned using LSM 780 NLO (Zeiss, Germany) with standard built-in emission-excitation spectra for each dye.

* *This reagent allowed us to make multiple series of LSM scans without significant decreasing of signal intensity. DABCO was diluted according to the methodology (25 mg / ml DABCO in 90% glycerin mixed with 10 % 1×PBS, pH = 8.6)*

**References**

Arumuganathan, K., & Earle, E. D. (1991). Nuclear DNA content of some important plant species. Plant molecular biology reporter, 9(3), 208-218.

Arumuganathan, K., & Earle, E. D. (1991a). Estimation of nuclear DNA content of plants by flow cytometry. Plant molecular biology reporter, 9(3), 229-241.

Harrington, B. J., & Hageage Jr, G. J. (2003). Calcofluor white: a review of its uses and applications in clinical mycology and parasitology. Laboratory medicine, 34(5), 361-367.

Kapuscinski, J. (1995). DAPI: a DNA-specific fluorescent probe. Biotechnic & Histochemistry, 70(5), 220-233.
