# Supplementary material for: LSM-W2: laser scanning microscopy worker for wheat leaf surface morphology
Source: BMC Syst Biol. 2019 Mar 5;13(Suppl 1):22. doi: 10.1186/s12918-019-0689-8 (PMC6399813; doi:10.1186/s12918-019-0689-8)
Supplement: Supplementary file 2 — LSM-W2 user manual. (DOCX 1266 kb) [file 12918_2019_689_MOESM2_ESM.docx]

**Additional file 2**

**LSM_W^2^ manual**

**Installation:** Download LSM_Worker.jar to the plugins folder, and restart ImageJ (Fiji), and there will be a new "LSM_Worker" command in the Plugins menu.

**Description**: The plugin implements an image-processing algorithm for detection the wheat leaf epidermis cellular structure from confocal laser scanning microscopy. The obtained 3D-images are multi-frame and multi-channel lsm-files and have the following features. First, the outer part of the cell wall of the epidermal cells has a higher signal intensity than the lower one. Secondly, trichomes of pubescent wheat varieties rise above the epidermal layer, and their cell wall also has a more intense signal than other epidermal cells. These peculiarities lead to unsatisfactory quality of 3D cell segmentation. At the same time, the used staining and scanning conditions lead to a proper segmentation of nuclei from 3D images for several layers of cells by the watershed algorithm. To improve the situation for cells segmentation, we developed a computer vision algorithm for detecting a leaf surface. This algorithm uses data on the signal intensity along the z-axis in the image obtained as a linear combination of two channels and constructs a projection of the cell walls of the epidermal cells on 2D-plane. Additional processing of the detected leaf surface by smoothing filters and morphological operators results in improving the quality of the projection and accurately segmenting of the cells by the watershed algorithm. We also use the defined leaf surface to isolate nuclei of the epidermal cells from others. The next step calculates the correspondence between the segmented cells and nuclei.


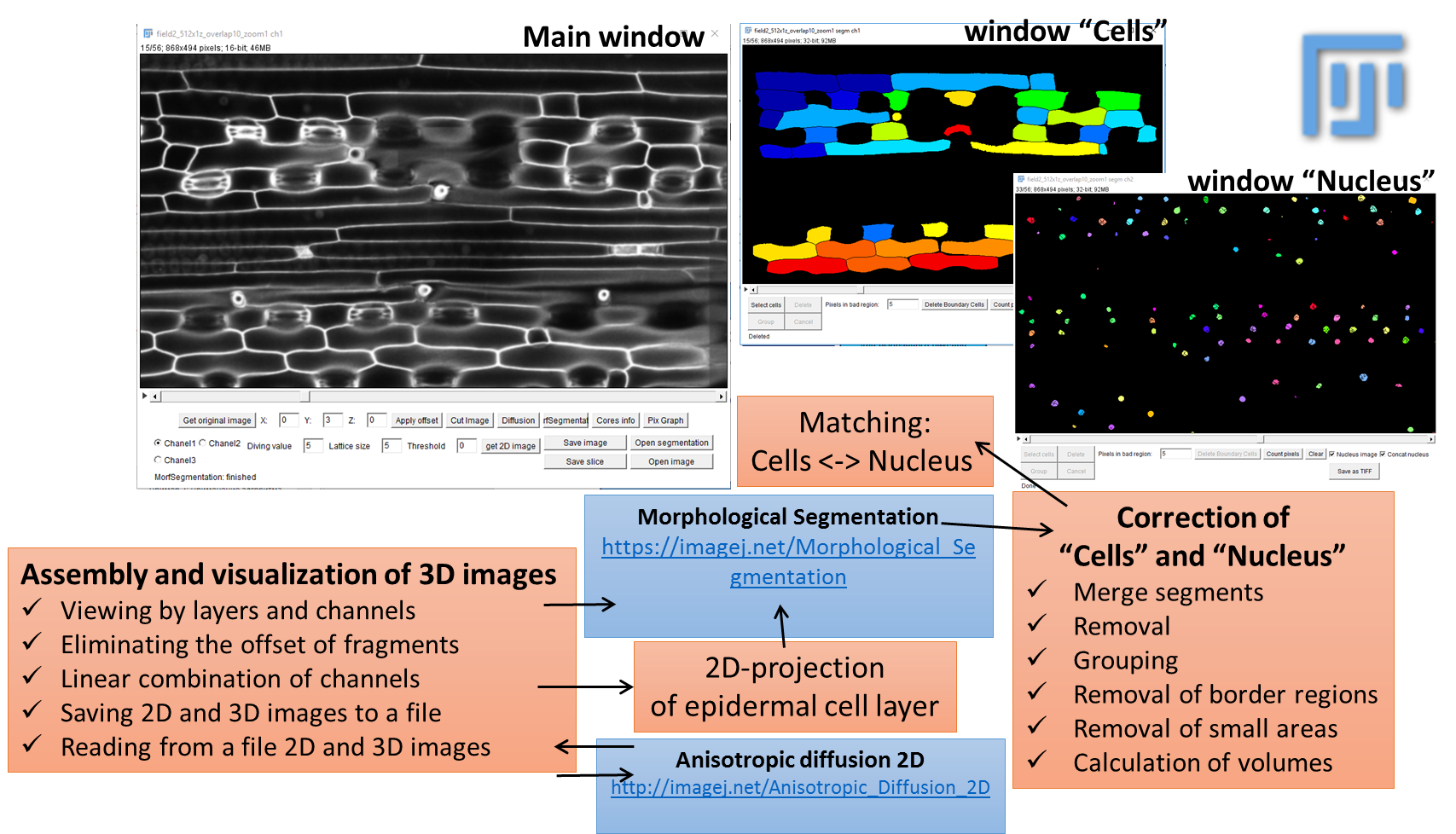


General view of the main working windows of LSM_W^2^

**Getting started**

After starting the plug-in will be asked to select an image file. The valid choice is only lsm-files.

As soon as the information from the file is read, a window will be displayed showing the necessary information to form a complete multi-frame multi-channel 3D image. To continue working with the plugin, one must specify:

- The number of fragments by X-axis and Y axis
- The overlap percentage
- If necessary, one can reduce the amount of memory consumed by compressing the image (reducing the size and depth of color). Choosing this option will result in data loss, and possible deterioration of the information received, but for stable operation of the plug-in, the memory consumption during the download phase should not exceed 50% of the available RAM.

After one specify all the necessary information, click "OK" and wait for the image formation.


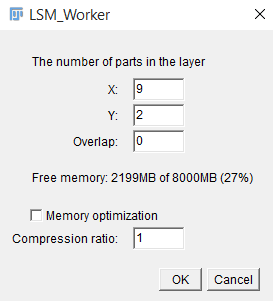


General view of the dialog box when opening images

**The main window of “LSM W^2^”**

The window header displays basic information about the image, including the current slice number and the total number of slices, the size of one slice in pixels and microns, the color depth, and the whole image size. In the central part of the main window, there is a layer-by-layer display of the image formed from the frames for the selected channel (the default is channel 1). At the bottom of the main window, there are buttons and fields for working with the image.


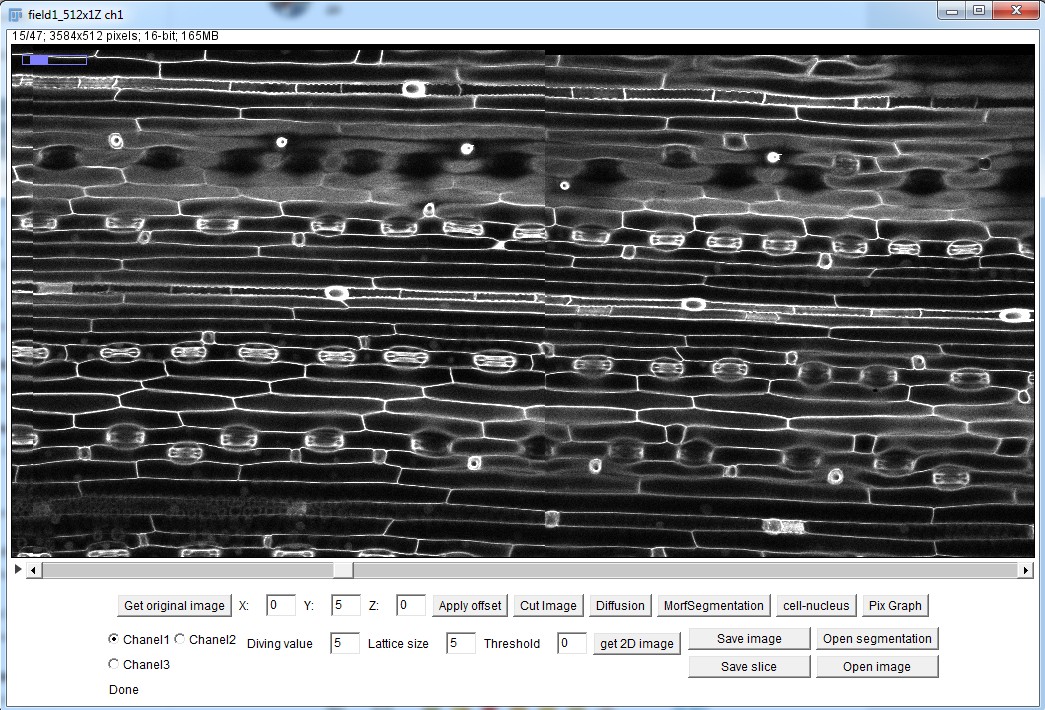


General view of the main window of the LSM_W^2^ plugin

Consider the functions of the buttons located on the first line:

- "Get original image" button cancels all changes and returns the original image.
- Button "Apply offset" produces successively shift of the frames to eliminate the offset. In the fields "X", "Y" and "Z", one can specify the number of pixels to which each image frame in the corresponding plane should be moved. Repeated use of this option shifts relative to the original image, rather than the current.
- The "Cut Image" button cuts the selected area for all channels from the source image. Before applying this function, you must select a rectangular fragment in the image using the standard ImageJ tool.
- The "Diffusion" button allows one to apply an anisotropic diffusion filter layer by layer for all layers of the selected channel. This button calls the plugin "Anisotropic diffusion 2D" (https://imagej.nih.gov/ij/plugins/anisotropic-diffusion-2d.html).
- The "MorfSegmentation" button calls a third-party module "Morphological segmentation" (http://imagej.net/Morphological_Segmentation) for segmentation of the selected channel. When this button is clicked, the main window will be closed until the "Morphological segmentation" plugin is completed (for the further information, see "The module of morphological segmentation using watersheds" section).
- The "Cells/Nucleus Info" button maps the segmented images of the cell walls and nucleus and displays the result in the table (for more details see the "Conclusion of the results of the comparison of cell walls and cores" section).
- The "Pix Graph" button allows one to plot the intensity change graph depending on the layer number ("depth" of the image). After clicking the button, one must point the image pixel by the mouse. This information may be required to visualize the influence of the parameter of the maximum "Threshold", which is used for the construction of 2D projection for the surface layer (see details below). This option helps to select this parameter and to study the specificity of the image.

The second line of the menu contains the following components:

- A radio button allows one to select the image channel and display it in the central part of the main window. The standard image contains two channels visualizing the channel for the image of the cell walls (channel 1) and the channel for the image of the cell nuclei (channel 2). The additional channel (channel 3) is generated artificially by calculating the difference between the first and second channels. Many functions, such as segmentation depend on the selected image channel.
- Area for specifying the parameters for constructing a 2D projection of the upper-level cell walls. The "Get 2D Image" button builds this projection depending on the specified parameters:
  - The Diving value indicates how much pixels should be descended from the surface layer found.
  - Lattice size specifies the number of pixels between adjacent nodes for the algorithm of construction and calculation of the surface layer.
  - The maximum "threshold" (Threshold) determines the optimal maximum point. The maximum point is called optimal if it is a local maximum and when moving toward it to the left (the intensity increases), the intensity change at a single step (dx = 1) is greater than the set parameter. If the last condition is not satisfied, the optimal point is the maximum.
- A block of buttons to load a new one and save the current image. Loading an image replaces the current image for the selected channel with a new one, for the first and second channel. After downloading, some options are disabled. Reloading an image places this image on the selected channel. Thus, it is possible to upload an image consecutively for each channel. All the original meta information is stored for the new image. This approach allows you to download images that belong to the source, in different formats. The correspondence of the images is not checked and lies on the conscience of the expert interacting with the plugin. The ability to save an intermediate stage of work, and then reopen the file and continue processing, is an additional functionality and is used in rare cases.
- The "Open segmentation" button opens the segmented image.
