# Supplementary material for: LSM-W2: laser scanning microscopy worker for wheat leaf surface morphology
Source: BMC Syst Biol. 2019 Mar 5;13(Suppl 1):22. doi: 10.1186/s12918-019-0689-8 (PMC6399813; doi:10.1186/s12918-019-0689-8)
Supplement: Supplementary file 3 — LSM-W2 class diagram. (JPG 401 kb) [file 12918_2019_689_MOESM3_ESM.jpg]

**About this supplement**

This article has been published as part of *BMC Systems Biology Volume 13 Supplement 1, 2019: Selected articles from BGRS\SB-2018: systems biology.* The full contents of the supplement are available online at https://bmcsystbiol.biomedcentral.com/articles/supplements/volume-13-supplement-1.
